# Early is on time: Minimizing implantable cardioverter-defibrillator shocks through expedited antitachycardia pacing

**DOI:** 10.1016/j.hroo.2025.07.023

**Published:** 2025-08-06

**Authors:** Steven Mullane, Camden Harrell, Valentina Kutyifa, Luigi Di Biase, Malini Madhavan, Gaurav A. Upadhyay, Jim W. Cheung

**Affiliations:** 1 Biotronik, Inc., Lake Oswego, Oregon; 2 School of Medicine and Dentistry, University of Rochester, Rochester, New York; 3 Cardiac Arrhythmia Center, Division of Cardiology, Department of Medicine, Montefiore-Einstein Center for Heart and Vascular Care, Albert Einstein College of Medicine, Bronx, New York; 4 Department of Cardiovascular Medicine, Mayo Clinic, Rochester, Minnesota; 5 Center for Arrhythmia Care, Section of Cardiology, The University of Chicago, Pritzker School of Medicine, Chicago, Illinois; 6 Division of Cardiology, Department of Medicine, Weill Cornell Medical College, NewYork-Presbyterian Hospital, New York, New York

**Keywords:** Implantable cardiac defibrillator, Ventricular tachycardia, Ventricular fibrillation, Antitachycardia pacing, Shock therapy, Device programming

**Key Findings**

▪ Early antitachycardia pacing (ATP) is a feature in newer generations of implantable cardiac defibrillators that delivers ATP earlier than does traditional ATP.
▪ A total of 8453 ventricular fibrillation (VF) episodes (1573 with early ATP and 6880 with traditional ATP) were evaluated in a real-world registry.
▪ Early ATP increased the termination rates for fast ventricular tachyarrhythmias detected within the VF zone compared with traditional ATP (70.9% vs 51.0%; *P* < .0001).

## Introduction

Antitachycardia pacing (ATP) is an effective programmable therapy in implantable cardiac defibrillator (ICD) devices.^1^ Using ATP as a first-line intervention for terminating stable ventricular tachyarrhythmias (VTs) can reduce the risks associated with delivering high-energy shocks, such as tissue damage, lead-related complications, and psychological impact on patients,^2^ but can only be done once the episode meets the ICD’s detection criteria. Newer generations of Biotronik ICDs offer early ATP OneShot (eATP), which delivers ATP therapy earlier—when 12 of 16 events and stability criteria are satisfied—to target fast VTs detected in the programmed ventricular fibrillation (VF) therapy zone. The potential benefits of eATP are understudied; therefore, we performed a real-world analysis comparing the success rates of eATP and traditional ATP OneShot (tATP) programming for device-detected episodes within the VF zone.

## Methods

Remote monitoring data from ICDs capable of eATP programming (n = 3267), with transmitted VF episodes within the CERTITUDE database,^3^ were analyzed. VF episodes were divided into the eATP or tATP group on the basis of VF zone programming. To ensure comparability, episodes with fewer than 16 of 20 detection events were excluded, as these do not meet the minimum criteria for eATP programming. Each episode in the VF zone was evaluated for whether ATP was delivered, whether the event self-terminated (without ATP or shock), or whether shock therapy was delivered without ATP. ATP was considered successful if no subsequent shock was required after ATP delivery. Device programming data, R-R intervals at detection, and episode duration for each episode were analyzed using descriptive statistics. This retrospective analysis was performed using real-world evidence methodologies and has been approved by an institutional review board, which granted a waiver of informed consent and a full waiver of Health Insurance Portability and Accountability Act authorization owing to the minimal risk and observational nature of the analysis. The research reported in this study adhered to the guidelines set forth by the Office of Human Research Protection, which is supported by the US Department of Health and Human Services.

## Results

**Figure 1:**
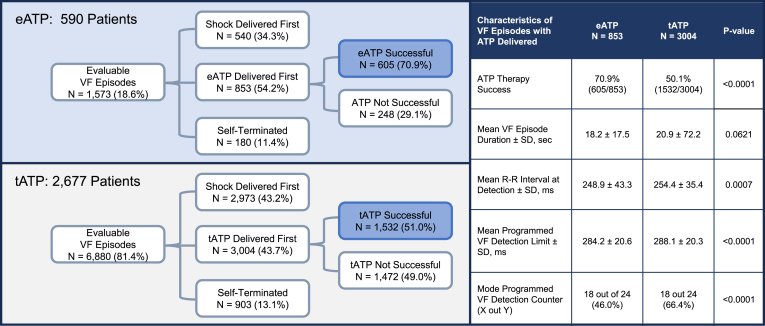
Comparison of early antitachycardia pacing (ATP) OneShot (eATP) and traditional ATP OneShot (tATP) for ventricular fibrillation (VF) episodes.

A total of 8453 evaluable VF episodes (1573 with eATP enabled [18.6%] and 6880 with tATP enabled [81.4%]) from 3237 patients were analyzed. The self-terminating rate was 11.4% for the eATP group compared with 13.1% for the tATP group (*P* < .0169) (Figure 1). Successful termination rates were significantly higher in eATP-enabled episodes than in tATP-enabled episodes (70.9% vs 51.0%; *P* < .0001). Despite the eATP group having a faster programmed VF detection threshold, the mean R-R interval at VF episode detection was similar across both groups (*P* = .88). Combining the rates of VF episodes with no shock therapy (self-termination with successful ATP), the eATP group avoided shocks in 49.9% of the episodes (785 of 1573) compared with 35.4% (2435 of 6880) in the tATP group.

## Discussion

The results suggest that early ATP significantly improves termination rates for fast VTs detected within the VF zone, potentially reducing shock therapies. This may translate to a reduction in “unnecessary” shocks—those delivered when the episode would either spontaneously terminate or be terminated by ATP—an important finding as reductions in inappropriate shock therapy is associated with a reduction in all-cause mortality.^4^ It may also extend ICD battery lifespan and decrease the need for device replacements.^5^^,^^6^ Although self-termination rates were lower in the eATP group, which might suggest some inflation in the eATP’s success rate, the similarities in episode characteristics imply that early intervention with eATP may prevent arrhythmia progression or promote organized rhythms more amenable to successful termination. As technology continues to advance, ATP therapy is becoming increasingly tailored and sophisticated, improving outcomes and quality of life for patients with cardiac rhythm disorders.

### Limitations

This retrospective observational analysis was limited to episodes that were transmitted via remote monitoring. Episode information can be transmitted by the device at the subsequent transmission opportunities; therefore, potential bias because of delayed transmission (eg, patient being evaluated at hospital after shock therapy) is considered minimal. As episode adjudication was not possible, this analysis relied solely on device-detected events. Patient characteristics, such as primary or secondary prevention indication, were not evaluated or controlled for, which could affect the likelihood of ATP successfully terminating the episode. Further investigation through a randomized controlled trial or collection or patient characteristics to allow propensity score matching would strengthen the evidence by eliminating potential group differences and bias introduced when programming patients to eATP vs tATP. Because of the data available for analysis, we were unable to evaluate differences in acceleration of VTs between the groups. Evaluating potential proarrhythmic differences between groups should be considered in future research.

## Conclusion

This real-world analysis demonstrated that earlier delivery of ATP using the eATP feature increased the success rate of terminating fast monomorphic VTs detected in the VF zone compared with traditional programming. These findings highlight the possibility of eATP to reduce the frequency of unnecessary shocks, providing a meaningful benefit for patients with ICDs.

## Disclosures

Mr Mullane and Mr Harrell are employed by Biotronik, Inc. Dr Kutyifa has received research grants from Boston Scientific, ZOLL, the National Institutes of Health, and Spire; speaker fees from Medtronic, Abbott, and Biotronik, Inc.; and consultant fees from Biotronik, Inc. Dr Di Biase serves as a consultant for Stereotaxis, Biosense Webster, Boston Scientific, Abbott Medical, and I-Rhythm and has received speaker honoraria/travel support from Medtronic, AtriCure, Biotronik, Inc., Baylis Medical, and ZOLL. Dr Upadhyay has received consulting fees and speaker honoraria from Abbott, Biotronik, Inc., Boston Scientific, GE Healthcare, Medtronic, Philips, Rhythm Science, and ZOLL. Dr Cheung has received research grants from Boston Scientific and has served as a consultant for Abbott, Biotronik, Inc., and Boston Scientific.

Drs Kutyifa, Di Biase, Madhavan, Upadhyay, and Cheung are members of the CERTITUDE Steering Committee, the research registry from which this manuscript was generated. As Steering Committee members, they are compensated by Biotronik, Inc. for their contributions and work performed for CERTITUDE.
